# Activation of persulfates by ferrocene–MIL-101(Fe) heterogeneous catalyst for degradation of bisphenol A

**DOI:** 10.1039/c8ra07007e

**Published:** 2018-10-29

**Authors:** Yu Wang, Weilin Guo, Xianghui Li

**Affiliations:** School of Water Conservancy and Environment, University of Jinan Jinan 250022 P. R. China chm_guowl@ujn.edu.cn

## Abstract

In this study, a novel and high-performance catalyst was prepared and used as the heterogeneous catalyst to activate persulfate for bisphenol A (BPA) degradation. Ferrocene was anchored to NH_2_-MIL-101(Fe) post-synthetically by the condensation of amine group from NH_2_-MIL-101(Fe) with the carbonyl group of ferrocenecarboxaldehyde. The synthesized ferrocene tethered MIL-101(Fe)–ferrocene was characterized by scanning electron microscopy, Fourier transform infrared spectroscopy, X-ray diffraction, X-ray photo-electron spectra, cyclic voltammetry and electrochemical impedance spectroscopy. The ferrocene acts as a redox mediator, which makes the ferrocene functionalized NH_2_-MIL-101(Fe) highly active in the degradation of BPA by accelerating the rate of the charge-transfer processes in aqueous solution. MIL-101(Fe)–Fc was proved to be the most effective catalyst, removing more than 99.9% of BPA. In addition, the catalyst can be reused without significant loss in activity.

## Abstract 2

In this study, a novel and high-performance catalyst ferrocene-MIL-101(Fe) was prepared and used as the heterogeneous catalyst to activate persulfate for bisphenol A degradation.

## Graphical abstract

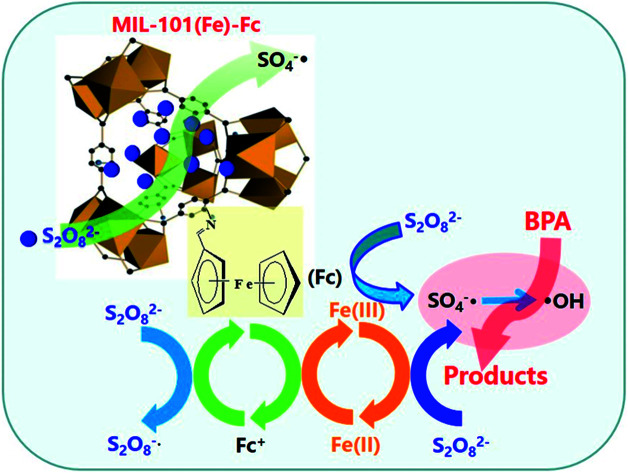

## 1. Introduction

Bisphenol A (2,2-bis(4-hydroxyphenyl)propane, BPA), as a typical environmental endocrine disruptors (EEDs), is extensively used in beverage and food containers, adhesives, flame retardants, and construction materials.^1^ The extensive use of BPA in manufactured products has resulted in its continuous release and distribution into the aquatic environment. This phenomenon can bring about bioaccumulation and reproductive toxicity, which has adverse effects on human health and the ecological environment.^2–5^ Advanced oxidation processes (AOPs) have been considered as a promising technology to treat water and wastewater containing toxic and non-biodegradable pollutants due to the generation of highly reactive radicals.

Sulfate radicals-based AOPs have drawn increasing attention. Recent studies have reported that sulfate radicals (SO_4_^−^˙) are more active and stable for oxidation than HO˙, and possess short reaction time and high utilization rate. There are various approaches of activation of peroxymonosulfate (PMS) or persulfate (PS) to produce SO_4_^−^˙, such as photolysis, electron transfer from transition metals, thermal activation, radiolysis and pyrolysis.^6–9^ Nie *et al.*^6^ proposed that the application of thermally activated persulfate for the removal of chloramphenicol. Oh *et al.*^9^ designed a novel quasi-cubic CuFe_2_O_4_–Fe_2_O_3_ catalyst to active peroxymonosulfate for the oxidation of BPA. However, these procedures have some notable drawbacks, like slow reaction processes, the overall lack of rate, high energy consumption, *etc*, which limit the development of these approaches. Therefore, it is critical to synthesize an alternative catalyst to overcome the aforementioned drawbacks.

Compared with the conventional catalysts, Fe-based catalysts exhibit captivating merits since they are earth-abundant, can be easily fabricated, and are almost non-toxic.^10^ Recent studies report that Fe-based catalysts present excellent catalytic performance in advanced oxidation systems,^11^ such as Fe_3_O_4_ nanoparticles, which have been applied as heterogeneous catalysts in persulfate activation for decomposing organic contaminants in aqueous solution.^12–14^ MIL-101(Fe), a typical Fe-based metal–organic frameworks (Fe-MOFs), has gained increasing attention for catalysis in recent years due to its structural flexibility, high surface area, massive porosity, and adjustable pore size. Barbosa *et al.*^15^ demonstrated that the MIL-101(Fe) and its amino-functionalized NH_2_-MIL-101(Fe) are both ideal catalytic materials in wastewater treatment. Li *et al.*^16^ proved that MIL-101(Fe) holds highly catalytic activity toward persulfate for the degradation of Acid Orange 7 (AO7). These studies comprehensively indicate that MIL-101(Fe) is a promising candidate for the activation of persulfate.

Ferrocene (Fc) is comprised of a central Fe^2+^ atom bounded to two cyclopentadienyl rings on opposite sides, forming a transition metallic compound. The electron donor–acceptor conjugated structure of Fc makes it of high redox reversible properties, showing a potential Fenton-like catalyst. Fc and immobilizing Fc on supports (such as silica, carbon materials, SBA-15, single-walled carbon nanotube, and so on) were used as a catalyst to establish a new Fc/Fenton system.^17,18^ By the way, ferrocene is widely used as effective mediators to transfer electrons in bioelectrocatalysis, electro-oxidants in electrosynthesis and probes in electrochemical detection.^19–21^ Nevertheless, it is rarely implemented as a redox mediator on the degradation processes of pollutants.

Herein, a Fenton-like catalyst consisting of Fc-functionalized MIL-101(Fe) is developed *via* amidation reaction of ferrocenecarboxaldehyde with NH_2_ group from NH_2_-MIL-101(Fe) for the first time. MIL-101(Fe)–Fc exhibits excellent Fenton-like activity and good stability for the degradation of pollutants in a wide pH range, demonstrated using bisphenol A (BPA) as model pollutants. The effects of different reaction conditions on the degradation of BPA were discussed in detail. A possible BPA degradation mechanism was also proposed.

## 2. Experimental

### 2.1 Materials

BPA (purity 99.5%), 2-aminobenzene-1,4-dicarboxylic acid (NH_2_-BDC) and ferrocenecarboxaldehyde were supplied by Sigma Aldrich. *N*,*N*-Dimethylformamide (DMF), iron(iii) chloride hexahydrate (FeCl_3_·6H_2_O), potassium persulfate (K_2_S_2_O_8_) and absolute ethanol were purchased from Sinopharm Chemical Reagent Co. Ltd., China. Ultrapure water was produced by a Millipore Mill-Q system.

### 2.2 Preparation of MIL-101(Fe)–ferrocene

The NH_2_-MIL-101(Fe) catalyst was synthesized through solvothermal procedure as reported elsewhere.^16^ 2.45 mmol of FeCl_3_·6H_2_O and 1.24 mmol of NH_2_-BDC were dispersed into 15 mL of DMF solution. The mixture was ultrasonicated for 10 min, and then transferred into a Teflon-lined stainless steel autoclave with a volume capacity of 20 mL. After being heated at 110 °C for 20 h, the precipitate was collected by centrifugation and washed with DMF, water, and ethanol. The product was then dried overnight at 60 °C.

According to the literature,^16^ about 0.1 g of NH_2_-MIL-101(Fe) (0.12 mmol) was immersed in 15 mL absolute ethanol and 0.1 mmol of ferrocenecarboxaldehyde was added under stirring at room temperature. Afterwards, the resultant solution was heated at 80 °C to reflux under magnetic stirring for 2 h. MIL-101(Fe)–Fc was finally obtained by centrifugal separation from solution and thoroughly washed with absolute ethanol, then subsequent dried at 60 °C overnight.

Powder X-ray diffraction (XRD) patterns were obtained on a Rigaku Dmax/Ultima IV diffractometer with monochromatized Cu Kα radiation (*λ* = 0.15418 nm).^22^ The chemical states of the composite were tested using X-ray photoelectron spectrometry measurements (XPS) and an ESCALAB 250 spectrometer (Thermo-VG Scientific) using Mg Kα radiation (1253.6 eV), while the binding energy values were calibrated with respect to C (1s) peak (284.6 eV). The microstructures of the samples were observed using scanning electron microscopy (SEM) on a XL30ESEM-TMP (Philips Electronics Co., Eindhoven, Netherlands). Fourier transform infrared (FTIR) spectroscopy was performed on Nicolet 6700 FTIR Spectrometric Analyzer in the range of 4000–500 cm^−1^. Electrochemical experiments were carried out on an electrochemical work station (CHI660E, Shanghai Chenhua Instruments, China), which was connected to a computer. The electrodes were assembled based on the method reported by Zhao *et al.*^23^ To prepare the NH_2_-MIL-101(Fe) and MIL-101(Fe)–Fc electrodes, the catalysts were added into absolute ethanol (10 mg mL^−1^) and ultrasonicated for 10 min, resulting in the formation of a homogeneous suspension. Subsequently, 0.3 mL of suspension was dropped onto the surface of the pretreated conducting indium tin oxide (ITO) glass and was plaed in an oven at 60 °C for 6–12 h. Electrochemical measurements were performed in a conventional three-electrode system with the NH_2_-MIL-101(Fe) or MIL-101(Fe)–Fc as the working electrode, a saturated calomel electrode (SCE) as the reference electrode, and a Pt wire as a counter electrode. The electrolyte for cyclic voltammetry (CV) and electrochemical impedance spectroscopy (EIS) was 0.1 mol L^−1^ KCl containing 5.0 mmol L^−1^ K_3_[Fe(CN)_6_] in aqueous solution.

### 2.3 Degradation experiments

The adsorption and catalytic tests were run in a 50 mL glass reactor for all experiments in duplicate. 60 mg L^−1^ BPA was prepared using deionized water. For the BPA degradation experiment, 5 mg of catalysts were dispersed in 25 mL 60 mg L^−1^ BPA aqueous solution without any oxidants, and the reaction mixture was magnetically stirred for 1 h to ensure the establishment of an adsorption–desorption equilibrium. This reaction was continued 2 h for thorough oxidation. After the oxidation reaction was started by adding 0.25 mmol persulfate, 5 mL of the suspension was collected periodically and immediately centrifuged at a speed of 8000 rpm for 1 min to separate the solid particles. High performance liquid chromatographic analysis (HPLC) was performed to measure BPA concentrations in the supernatant.

The concentration of BPA in the supernatant was analyzed on a Waters HPLC equipped with an Atlantis TC-C18 column (4.6 × 250 mm, 5 μm) at a UV wavelength of 276 nm. The mixed solvent of acetonitrile and water (50/50, v/v) was used as the mobile phase with a flow speed of 1 mL min^−1^. The integrated peak areas correlate with the BPA concentration. The quantitative analysis was done by external calibration with standards. Each analysis was repeated three times to ensure reliable results. The limit of quantification (LOQ) was found to be 34.00 μg L^−1^.

## 3. Results and discussion

### 3.1 Characterization

**Fig. 1:**
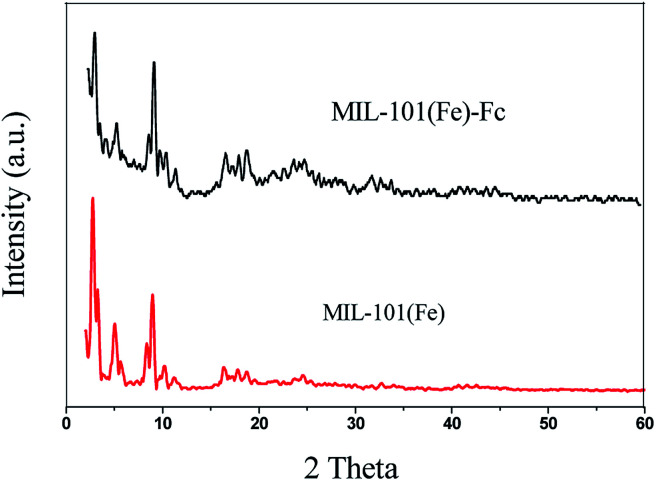
XRD patterns of MIL-101(Fe) and MIL-101(Fe)–Fc.

X-ray powder diffraction (XRD) patterns of MIL-101(Fe) and MIL-101(Fe)–Fc are depicted in Fig. 1. The diffraction peaks at 2*θ* of 2.75, 5.03, 8.36 and 8.96 demonstrates high quality of crystallinity of MIL-101(Fe), which corresponds to the previously reported one.^24^ Additionally, the XRD pattern of MIL-101(Fe)–Fc exhibits the presence of the characteristic peaks of MIL-101(Fe), except for at a lower intensity. Moreover, there is no other diffraction peaks appearing for MIL-101(Fe)–Fc, which suggests that anchoring ferrocene to MIL-101(Fe) has no influence on the structure of MIL-101(Fe).

**Fig. 2:**
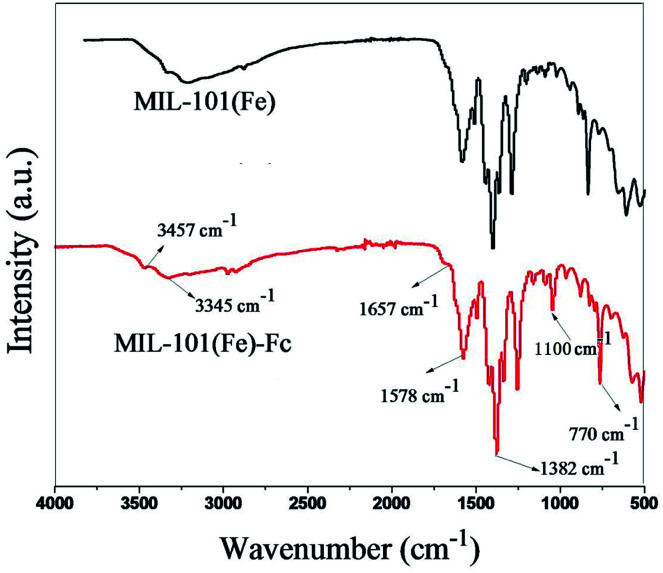
FTIR spectra of MIL-101(Fe) and MIL-101(Fe)–Fc.

The FTIR spectra of the MIL-101(Fe) and MIL-101(Fe)–Fc samples are shown in Fig. 2. Similar to MIL-101(Fe), characteristic bands of carboxylate group vibrations at 1300–1700 cm^−1^ and benzene ring at 770 cm^−1^ are found in MIL-101(Fe)–Fc.^16^ Two noticeable peaks can be found at 3345 and 3457 cm^−1^, which were derived from the asymmetrical and symmetrical stretching vibration modes of amine group in MIL-101(Fe), respectively. However, the intensity of two peaks in MIL-101(Fe)–Fc is markedly decreased in comparison with that of MIL-101(Fe), suggesting that the amine group of aminoterephthalic acid in MIL-101(Fe) had reacted with the aldehyde group of Fc-CHO. Simultaneously, the peak at about 1657 cm^−1^ can be ascribed to –CONH bands (mainly stretching of the C=O bonds).^25^ In addition, a peak can be observed at 1100 cm^−1^ in the IR spectrum of Fc-MIL, attributed to the C–H in-plane bending mode.^26^ All the results above indicate that the Fc moiety is successfully grafted into MIL-101(Fe).

**Fig. 3:**
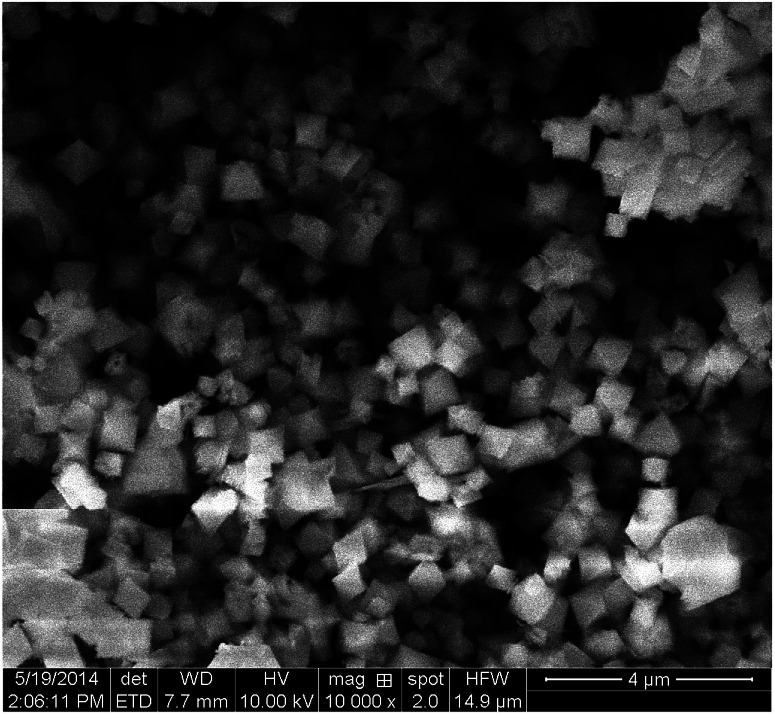
SEM image of MIL-101(Fe)–Fc.

The morphological details of these samples were analyzed by SEM. As shown in Fig. 3, MIL-101(Fe)–Fc has an unusual octahedron morphology and average diameter of 800 nm, similar to the results of the MIL-101(Fe) that Li *et al.* previously reported.^16^ This finding suggests that the ferrocene modification process does not noticeable damage the structure of MIL-101(Fe)–Fc.

**Fig. 4:**
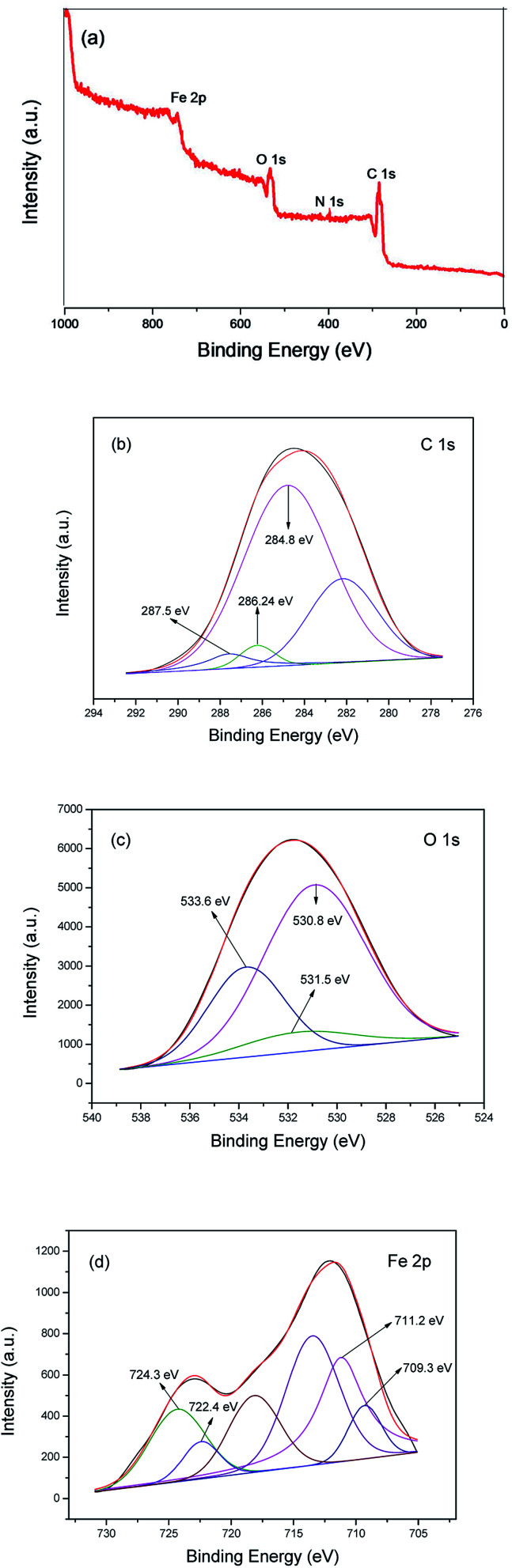
XPS spectra of MIL-101(Fe)–Fc: survey spectrum (a), C 1s (b), O 1s (c) and Fe 2p (d).

X-ray photoelectron spectroscopy (XPS) measurements were carried out to confirm the surface composition and analyze the chemical states of the elements in MIL-101(Fe)–Fc. The results (survey and high-resolution spectra) are presented in Fig. 4, which shows a wide survey scan taken in the range 0–1000 eV, and the wide scan XPS spectra of the MIL-101(Fe)–Fc shows photoelectron lines at a binding energy of around 285, 530, and 711 eV attributed to C 1s, O 1s, and Fe 2p, respectively. It also can be seen from Fig. 4a that the as-prepared composite contained four elements of C, O, N and Fe. In the spectrum of C 1s (Fig. 4b), the binding energy with peak at 284.8 eV is the characteristic of the combination of C–C, C=C and C–H bonds.^27,28^ The peak of C=O bonds is located at 287.5 eV,^29^ while the peak at 286.24 eV is attributed to C=N bonds, suggesting that ferrocene was anchored to NH_2_-MIL-101(Fe) post-synthetically through the condensation of the amine group from NH_2_-MIL-101(Fe) with the carbonyl group of ferrocenecarboxaldehyde. Fig. 4c presents the high-resolution XPS spectrum of the O 1s. The wide and asymmetric O 1s XPS peak implied that there was more than one chemical state. The peak at 530.8 eV is associated with Fe–O. The peak at 531.5 eV corresponded to surface hydroxyl groups (O–H), while the peak located at 533.6 eV corresponded to the carboxylate groups of the BDC linkers (C=O).^28,30,31^ Measuring the Fe 2p spectrum aides in understanding the chemical state change of Fe in the product. The Fe 2p XPS spectrum of MIL-101(Fe)–Fc is shown in Fig. 4d, which illustrates the Fe 2p3/2 and Fe 2p1/2 binding energy peaks centered at 711.4 eV and 721.2 eV, respectively. These peaks originated from Fe^3+^ in MIL-101(Fe)–Fc, as reported by Li *et al.*^16^ There are also two peaks around binding energy of 709.3 and 722.4 eV attributed to Fe^2+^, as expected for the divalent state of Fe in ferrocene, further implying that MIL-101(Fe) had successfully modified with ferrocene.

**Fig. 5:**
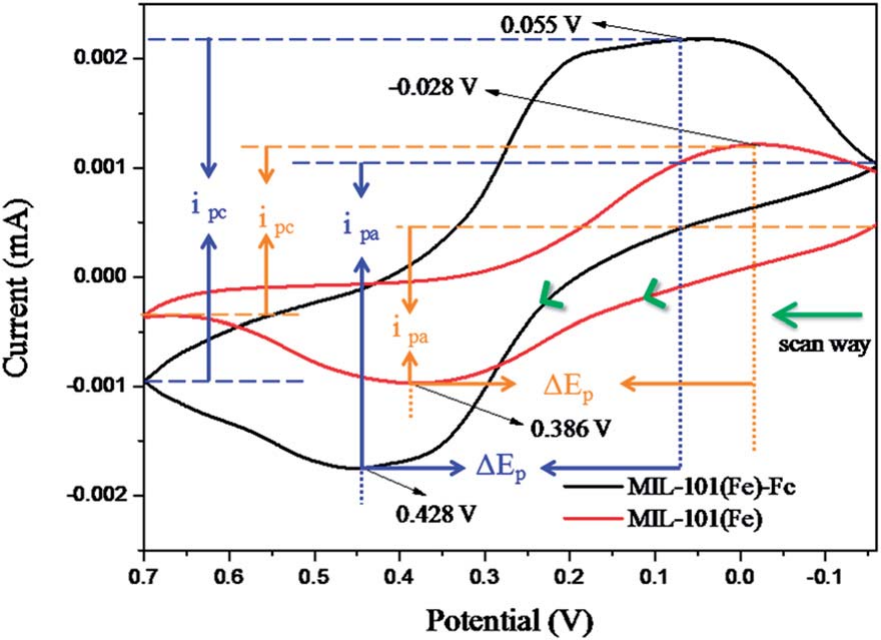
Cyclic voltammetric (CV) responses at a scan rate of 50 mV s^−1^ using MIL-101(Fe) or MIL-101(Fe)–Fc, respectively (Δ*E*_p_, peak potential difference, *i*_pa_, anodic current, *i*_pc_, peak cathodic).

Fig. 5 shows the electrochemical performances of as-prepared samples in 0.1 mol L^−1^ KCl solution containing 5.0 mmol L^−1^ K_3_[Fe(CN)_6_]. In the Fig. 5, compared with MIL-101(Fe), MIL-101(Fe)–Fc responds at higher currents and the peak separations decrease slightly. The peak currents at MIL-101(Fe)–Fc (*i*_pa_ = −2.75 μA, *i*_pc_ = 3.1 μA) essentially double in comparison to the peak currents at MIL-101(Fe) (*i*_pa_ = −1.45 μA, *i*_pc_ = 1.55 μA). While the peak separations at MIL-101(Fe)–Fc decreased modestly, the Δ*E*_p_ for MIL-101(Fe)–Fc and MIL-101(Fe) are 373 mV and 414 mV, respectively. These findings suggest that the presence of ferrocene in nanocomposites can accelerate electron transfer.^32^ Based on the analysis above, it can be concluded that MIL-101(Fe)–Fc is superior to MIL-101(Fe) in the electrocatalytic activity. This result is consistent with previous publications.^33^

### 3.2 Adsorption and catalytic ability

**Fig. 6:**
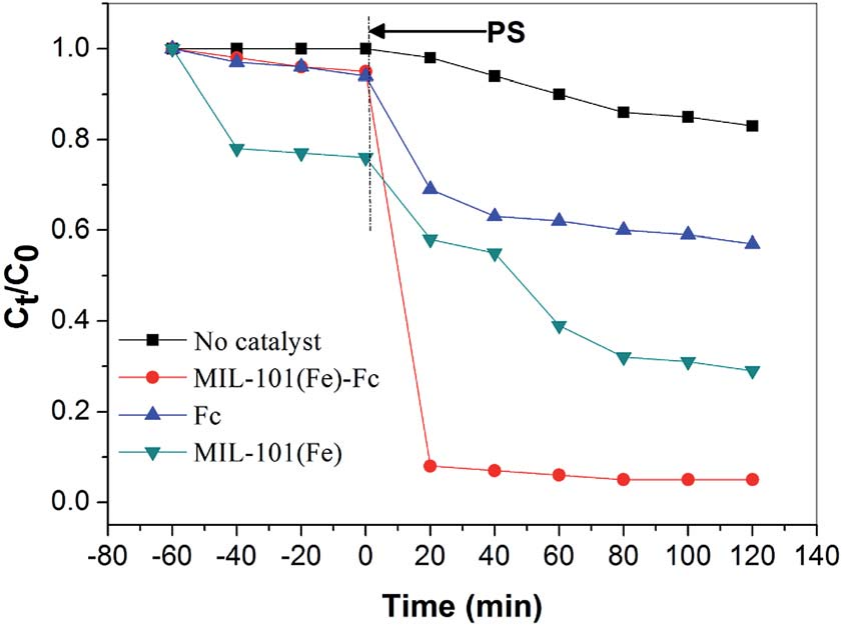
Degradation of BPA with different catalytic conditions (BPA: 60 mg L^−1^, catalyst: 0.2 g L^−1^, persulfate: 10 mmol L^−1^, temperature: 25 °C, and initial pH: 5.76).

The degradation of BPA was analyzed to evaluate and compare the adsorption and catalytic performance on MIL-101(Fe)–Fc and MIL-101(Fe). Fig. 6 illustrates the time dependence of the BPA removal over different catalysts. The resulting data shows that BPA adsorption on the materials mentioned above is a relatively brief process, that reaches the adsorption/desorption equilibrium in around 40 min. Fig. 6 also indicates that prior to oxidative treatment, the adsorption amount of BPA by MIL-101(Fe)–Fc is obviously lower than that of MIL-101(Fe), 5% and 30%, respectively. This is because the bulkier functional groups (such as –Fc) introduced in MIL-101(Fe) presented a greater reduction in the surface area.

When persulfate was further introduced into the system, a sharp increase in the degradation of BPA was subsequently observed, and the catalytic degradation capacity of BPA over MIL-101(Fe)–Fc proved to be evidently much higher than that of MIL-101(Fe). BPA was nearly completely removed within 40 min in the MIL-101(Fe)–Fc/PS process. These findings further illustrate that the anchored ferrocene plays an important part in the electrons transfer for the catalytic degradation of BPA.

In addition, persulfate itself oxidized about 18% BPA within the examined time. In this study, Fc/PMS has also been investigated for pollutant degradation. For pure Fc, 41.3% BPA removal efficiency was achieved. This is because Fc can catalyze persulfate to produce SO_4_^−^˙ enhancing the degradation of BPA.

### 3.3 Mechanism

**Fig. 7:**
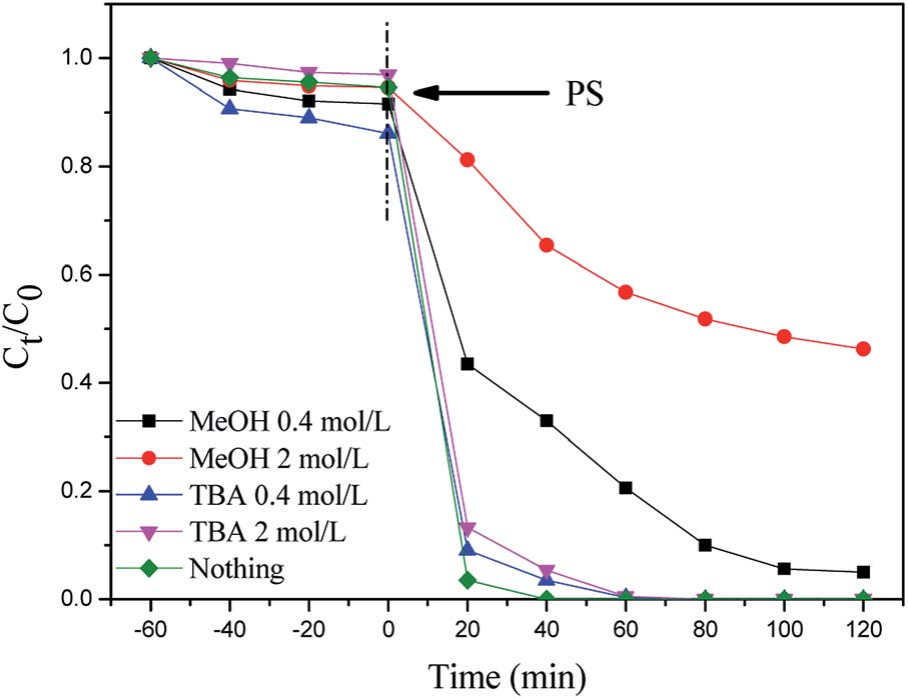
Effect of methanol and TBA on the degradation of BPA (BPA: 60 mg L^−1^, catalyst: 0.2 g L^−1^, persulfate: 10 mmol L^−1^, temperature: 25 °C, and initial pH: 5.76).

In the above experiments, MIL-101(Fe)–Fc is proved to be an effective activator for the generation of reactive oxidants by persulfate. To identify the key factor responsible for the accelerated catalytic degradation of BPA in the MIL-101(Fe)–Fc/PS process, active species trapping experiments have been conducted. As is well known, methanol is extensively employed as a scavenger of hydroxyl and sulfate radicals, while tertiary butyl alcohol (TBA) is an effective quenching agent for hydroxyl radicals. Fig. 7 displays the BPA reduction results in the presence of different trapping agents. These results demonstrate that 99% of BPA could be degraded without any scavenger after 100 min. It can be found that the introduction of methanol caused the degradation rate of BPA to decrease from 99.9% to 70% after 100 min. When the concentration of methanol increased to 2 mol L^−1^, the degradation rate of BPA rapidly dropped to approximately 35% in the same period. However, the sulfate radicals quenching effect for TBA was minimal, indicating that the sulfate radicals are the dominant active species in the catalytic process.

**Fig. 8:**
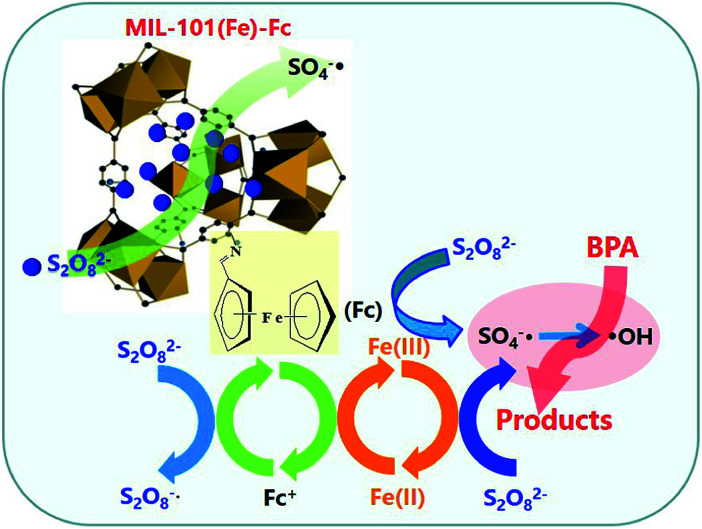
A schematic illustration of catalytic oxidation of BPA over MIL-101(Fe)–Fc.

Based on the results above, Fig. 8 presents a plausible mechanism for the degradation of BPA over MIL-101(Fe)–Fc in the presence of persulfate. There are two key active mechanisms in the degradation of BPA that must be considered, on one hand, the active metal sites Fe^3+^ on the porous MOFs can strongly react with oxidant molecules in catalytic process,^16^ which activate persulfate to produce a great quantity of S_2_O_8_^−^˙ and Fe^2+^ (eqn (1)). Then the generated Fe^2+^ is oxidized by persulfate to form SO_4_^−^˙ according to eqn (2). Meanwhile, ferrocene can act as an electron shuttle when tethered to MIL-101(Fe) and accelerate the process of electron transfer through its reversible oxidization and reduction. On the other hand, ferrocene conducts self-redox cycling to produce SO_4_^−^˙ as depicted by eqn (3) and (4). The double pathways greatly promote the net SO_4_^−^˙ production, thereby significantly accelerating the degradation of BPA. Moreover, SO_4_^−^˙ radicals are the main reactive species responsible for decomposing targeted contaminants in aqueous solution. Additionally, HO˙ can be generated *via* following reactions (eqn (5) and (6)), which also has effects on removing BPA.

Through these processes, the heterogeneous oxidation reaction experiences the transformation of Fe(iii)–Fe(ii) through eqn (1) and (2). Ferrocene acts as a redox mediator, improving the rate of the charge-transfer processes in the aqueous solution, shortening the reaction time, and significantly enhancing the removal rate of BPA.

1 Fe^III^ + S_2_O_8_^2−^ → Fe^II^ + S_2_O_8_^−^˙

2 Fe^II^ + S_2_O_8_^2−^ → Fe^III^ + SO_4_^−^˙ + SO_4_^2−^

3 Fc + S_2_O_8_^2−^ → Fc^+^ + SO_4_^−^˙ + SO_4_^2−^

4 Fc^+^ + S_2_O_8_^2−^ → Fc + S_2_O_8_^−^˙

5 SO_4_^−^˙ + H_2_O → HO˙ + H^+^ + SO_4_^2−^

6 SO_4_^−^˙ + OH^−^ →SO_4_^2−^ + HO˙

### 3.4 Stability and reusability

From a practical point of view, durability and potential reuse of the catalyst is an important characteristic when considering its application. To assess the stability and reusability of the as-prepared catalysts, the degradation rate of BPA was measured in the repeated experiments by recycling the used catalyst. After each degradation was brought to completion, the used catalysts were separated from the solution, washed with deionized water and ethanol several times to remove the BPA on its surface, and dried at 60 °C for 12 h. The repeated degradation was conducted three times with equal catalyst dose and other reaction conditions for the subsequent runs.

**Fig. 9:**
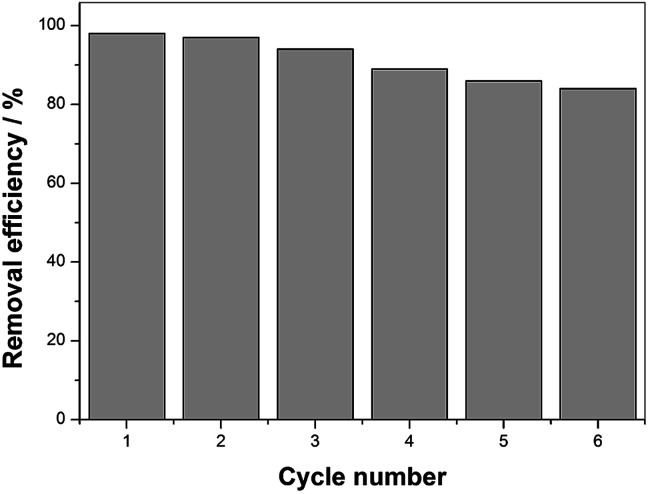
Catalytic property of MIL-101(Fe)–Fc for repeated use (BPA: 60 mg L^−1^, catalyst: 0.2 g L^−1^, PS: 10 mmol L^−1^, temperature: 25 °C, and initial pH: 5.76).

According to Fig. 9, the BPA removal remained above 80% after the sixth degradation procedure, suggesting MIL-101(Fe)–Fc can be recycled and reutilized for at least six successive cycles with an acceptable rate. However, the BPA removal rate displayed a slight decrease during the six consecutive cycles. The lower BPA degradation rate can be attributed to the one or both following reasons: (1) due to the small particle size, catalyst loss was unavoidable in the process of washing and drying, or (2) the adsorbed targeted contaminants on the surface of catalyst inhibited the interaction of catalyst and BPA. Based on the characteristics discussed above, the MIL-101(Fe)–Fc composite exhibits not only an excellent catalytic performance but also efficient recyclability, which is especially crucial for practical application.

## 4. Conclusions

In this study, we successfully synthesized a novel MIL-101(Fe)–Fc catalyst by a two-step method and studied its characteristics with several techniques. Ferrocene was anchored to NH2-MIL-101(Fe) post-synthetically by the condensation of amine group from NH2-MIL-101(Fe) with the carbonyl group of ferrocenecarboxaldehyde. The experimental results show that the as-obtained MIL-101(Fe)–Fc has higher catalytic activity than pure MIL-101(Fe). Controlled experiments using different trapping agents proved that SO_4_^−^˙ played the most prominent role in the degradation process. A reasonable mechanism for the enhanced catalytic activity was subsequently proposed by harnessing ferrocene as a redox mediator to promote the electrons transfer in the MIL-101(Fe)–Fc/PS process for the degradation of BPA. Furthermore, the MIL-101(Fe)–Fc catalyst holds the advantages of good reproducibility and outstanding stability. This work offers a new approach in the construction of high performance nanocomposite catalysts with improved electrons transfer for environmental remediation.

## Conflicts of interest

There are no conflicts to declare.

## Supplementary Material
